# Rapid clinical improvement of amyloid A amyloidosis following treatment with tocilizumab despite persisting amyloid deposition: a case report

**DOI:** 10.1186/s12882-017-0799-8

**Published:** 2017-12-29

**Authors:** Akira Yamagata, Takahiro Uchida, Yuji Yamada, Takashi Nakanishi, Kazue Nagai, Toshihiko Imakiire, Naoki Oshima, Hiroo Kumagai

**Affiliations:** 1 Department of Nephrology and Endocrinology, National Defense Medical College, 3-2 Namiki, Tokorozawa, Saitama 359-8513 Japan; 2 Department of Rheumatology, National Defense Medical College, Tokorozawa, Saitama Japan

**Keywords:** Amyloidosis, Nephrotic syndrome, Rheumatoid arthritis, Tocilizumab

## Abstract

**Background:**

Amyloid A amyloidosis is one of the most common forms of amyloidosis. It is secondary to rheumatoid arthritis, which is difficult to manage and has a poor prognosis. We present a patient with rheumatoid arthritis and amyloid A amyloidosis who was treated with tocilizumab, a humanized monoclonal antibody against interleukin 6 receptor, resulting in improvement in both proteinuria and gastrointestinal symptoms; however, amyloid deposition remained.

**Case presentation:**

A 67-year-old woman who had previously been treated for rheumatoid arthritis presented with abdominal pain and diarrhea. Right renal cell carcinoma was found, and amyloid A amyloidosis was diagnosed concomitantly based on colon biopsy. The renal cell carcinoma was resected, and the non-cancerous part of the renal tissue also showed amyloid A deposition. Following surgery, protein levels in the urine increased to the nephrotic range, and administration of tocilizumab was initiated, which resulted in resolution of the proteinuria. The patient’s gastrointestinal symptoms were also alleviated. However, repeat colon biopsy showed amyloid deposition.

**Conclusions:**

This case of amyloid A amyloidosis suggests that amyloid deposition indicates only structural change of the affected tissue, and that it is not amyloid deposition per se that causes the clinical symptoms of amyloidosis.

## Background

Amyloid A (AA) amyloidosis is one of the most common forms of amyloidosis, which is caused by the tissue deposition of serum amyloid A (SAA) protein, a major acute phase reactant protein [1, 2]. AA amyloidosis is often induced by a number of chronic inflammatory diseases, such as rheumatoid arthritis (RA) and chronic infections. AA amyloidosis secondary to RA is difficult to manage and the prognosis of patients with RA and AA amyloidosis is poor [3].

Biological agents are now widely used for the treatment of RA. In addition, they have recently been reported to be effective for the treatment of AA amyloidosis [4–7]. Tocilizumab (TCZ), a humanized monoclonal antibody that competitively inhibits the binding of interleukin-6 (IL-6) to its receptor [4], is one such agent. Although it has not been approved for the treatment of AA amyloidosis in Japan, disappearance of amyloid deposition in patients with RA and AA amyloidosis with TCZ treatment has been reported [4–7].

We, herein, present a case of RA and AA amyloidosis treated with TCZ, resulting in improvement in both proteinuria and gastrointestinal symptoms; however, amyloid deposition remained.

## Case presentation

A 67-year-old woman who had been previously treated for RA at a medical clinic presented with abdominal pain and chronic diarrhea, and was referred to our Internal Department. Her vital signs were normal, but pedal edema was present. The remainder of the physical examination findings were unremarkable.

Laboratory values were as follows: serum creatinine, 0.47 mg/dL; urea nitrogen, 10 mg/dL; serum total protein/albumin, 5.2/2.0 g/dL; immunoglobulin (Ig) G, 1002 mg/dL; IgA/M, 111/69 mg/dL. C-reactive protein (CRP), 4.9 mg/dL; rheumatoid factor, 38 IU/mL (reference range, < 15 IU/mL); anti-cyclic citrullinated peptide antibody, 124 U/mL (reference range, < 4 U/mL); matrix metalloproteinase-3, 248.4 ng/mL (reference range, 17.3 ~ 59.7 ng/mL); SAA, 193.2 μg/mL (reference range, < 8 μg/mL). Antinuclear antibody was undetectable, and urinary protein excretion was 0.5 g per gram urinary creatinine.

**Fig. 1:**
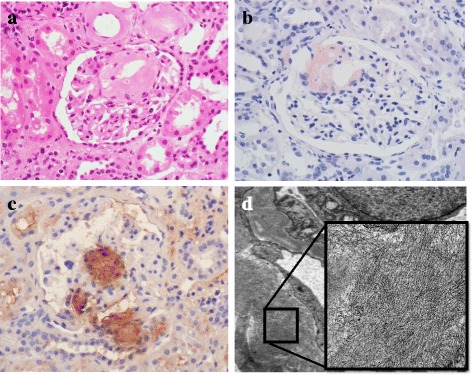
Histological features of the non-cancerous renal tissue. **a** Deposition of amorphous eosinophilic material was observed at vascular poles and mesangial regions (hematoxylin-eosin stain). The samples were both Congo red- (**b**) and amyloid A-positive (**c**). **d** Randomly dispersed, rigid, nonbranching fibrils were evident on electron microscopy. The width of the fibrils was 8 to 15 nm, and their length was 300 to 1000 nm (original magnification, **a**, **b**, **c**, ×400)

A computed tomography scan showed a right renal tumor suggestive of renal cell carcinoma. Right nephrectomy (May, 20XX) was performed and confirmed the diagnosis of clear cell carcinoma. The non-cancerous part of the renal tissue contained 365 glomeruli; 57 glomeruli were globally sclerotic. In addition, deposition of amorphous eosinophilic material was observed at vascular poles and mesangial regions (Fig. 1a). These deposits were Congo red- and AA-positive (Fig. 1b and c). Tubular atrophy and interstitial fibrosis were moderate. Immunofluorescence staining showed no deposition of complements or Igs, including light chains. Electron microscopy revealed randomly disposed, rigid, nonbranching fibrils compatible with amyloid deposition (Fig. 1d) and effacement of podocyte foot processes.

**Fig. 2:**
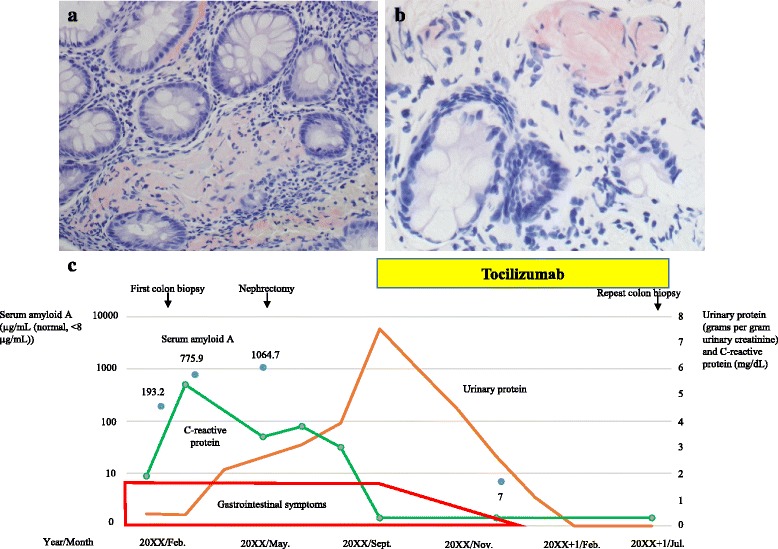
Persistent amyloid deposition on colon biopsy and the therapeutic course. **a** The first colon biopsy showed positive staining with Congo red at mucosal muscle plates and lamina propria. **b** The second biopsy showed that the amyloid deposition remained. **c** The patient’s clinical course shows that the gastrointestinal symptoms were alleviated and proteinuria gradually resolved after TCZ was initiated (original magnification, **a**, ×260, **b**, ×520)

Colonoscopy revealed amyloid deposition in addition to inflammation of the colon (February, 20XX, Fig. 2a). Biopsy of the duodenum also revealed amyloid deposition. The patient was therefore diagnosed with renal cell carcinoma and AA amyloidosis.

We initiated treatment with salazosulfapyridine for RA, but her proteinuria gradually increased to the nephrotic range (Fig. 2c). Salazosulfapyridine was replaced with TCZ, and her proteinuria began to improve considerably and eventually resolved (February, 20XX + 1). Her gastrointestinal symptoms were also alleviated. In addition, the levels of SAA decreased to the reference range. However, repeat colon biopsy (July, 20XX + 1), performed after her gastrointestinal symptoms resolved (November, 20XX), showed persistence of amyloid deposition, although it had decreased compared to that observed on the initial biopsy (Fig. 2b).

## Discussion and conclusions

In the present case report, treatment with TCZ induced rapid improvement of proteinuria and gastrointestinal symptoms associated with AA amyloidosis. SAA, the deposition of which is believed to cause AA amyloidosis, is synthesized by stimulation of proinflammatory cytokines, such as IL-1, IL-6, and tumor necrosis factor alpha [4]. Suppression of inflammation is therefore considered to be one of the most effective treatments to reduce the synthesis of SAA and to prevent the progression of amyloidosis.

Some cases of AA amyloidosis caused by renal cell carcinoma have been reported [8–10]; in such cases, improvement in amyloidosis was achieved after removal of the carcinoma. However, levels of CRP, an acute-phase protein used as a tumor marker of renal cell carcinoma, did not change in our patient and proteinuria increased after nephrectomy. These levels ultimately improved after TCZ treatment (Fig. 2c). This clinical course in our patient clearly showed that her amyloidosis was due to RA rather than renal cell carcinoma.

Recently, it has also been reported that TCZ ameliorated amyloid deposition in patients with AA amyloidosis [4–6]. However, in the current case, although proteinuria and gastrointestinal symptoms rapidly resolved with TCZ, a repeat colon biopsy showed that amyloid deposition remained. The amount of amyloid possibly has an impact on the patient’s symptoms. However, despite improvement of proteinuria in all patients treated with an anti-IL-1 receptor antagonist, renal amyloid deposition did not improve in one patient and even progressed in two patients [7], and amyloid deposition in the kidneys was reported to be more difficult to regress than that in the gastrointestinal tract [4]. Although we could not reassess the patient’s renal histopathology because of nephrectomy, it is possible that her proteinuria resolved without regression of renal amyloid deposition.

Selectivity index (SI) of proteinuria is related to the response to therapy in patients with nephrotic syndrome. For example, most patients with minimal change disease (MCD) who have a favorable response to therapy [11] show highly selective proteinuria. The renal prognosis of patients with AA amyloidosis has been poor, and it has also been reported that the SI of proteinuria in these patients was low [12]. However, our preliminary research showed that all four patients with biopsy-proven renal amyloidosis surprisingly showed highly selective proteinuria (Table 1). Although we did not evaluate the SI of proteinuria in the present case, it is possible that at least some patients with renal amyloidosis show highly selective proteinuria and that the renal prognosis of such patients is not poor if treated adequately.

**Table 1:** Summary of urinary protein and selectivity index in patients with renal amyloidosis at our Nephrology Department

Age	Urinary protein (grams per day)	Selectivity index
66-70	5.21	0.07
61-65	5.88	0.04
46-50	1.67	0.07
66-70	5.64	0.08

An experimental model of MCD showed that IL-6 was upregulated, and proteinuria in the model decreased in association with decreased IL-6 level [13]. IL-6 blockade has also been reported to decrease proteinuria in patients with AA amyloidosis [14–16]. These reports suggest that IL-6 could play a common pathogenic role in both MCD and AA amyloidosis. Further use of TCZ is therefore warranted for the treatment of AA amyloidosis, although investigation will be required in the future to evaluate this hypothesis.

In conclusion, we report a case of RA and AA amyloidosis in which amyloid deposition remained even after clinical symptoms of amyloidosis improved. This case suggests that amyloid deposition represents only structural changes in the affected tissue, and that it is not amyloid deposition per se that causes clinical symptoms of amyloidosis.

## Abbreviations

AA: Amyloid A
CRP: C-reactive protein
IgG: Immunoglobulin G
IL-6: Interleukin-6
MCD: Minimal change disease
RA: Rheumatoid arthritis
SAA: Serum amyloid A
SI: Selectivity index
TCZ: Tocilizumab

## References

[1] Falk RH, Comezo RL, Skinner M (1997). The systemic amyloidosis. N Engl J Med.

[2] Obici L, Raimondi S, Lavetelli F, Bellotti V, Merlini G (2009). Susceptibility to AA amyloidosis in rheumatic disease: a critical overview. Arthritis Rheum.

[3] Nakamura T (2011). Amyloid a amyloidosis secondary to rheumatoid arthritis: pathophysiology and treatment. Clin Exp Rheumatol.

[4] Matsui M, Okayama S, Tsushima H, Samejima K, Kanki T, Hasegawa A (2014). Therapeutic benefits of tocilizumab vary in different organs of a patient with AA amyloidosis. Case Rep Nephrol.

[5] Miyagawa I, Nakayamada S, Saito K, Hanami K, Nawata M, Sawamukai N (2014). Study on the safety and efficacy of tocilizumab, an anti-IL-6 receptor antibody, in patients with rheumatoid arthritis complicated with AA amyloidosis. Mod Rheumatology.

[6] Inoue D, Arima H, Kawanami C, Takiuchi Y, Nagano S, Kimura T (2010). Excellent therapeutic effect of tocilizumab on intestinal amyloid a deposition secondary to active rheumatoid arthritis. Clin Rheumatol.

[7] Topaloglu R, Batu ED, Orhan D, Ozen S, Besbas N (2016). Anti-interleukin 1 treatment in secondary amyloidosis associated with autoinflammatory diseases. Pediatr Nephrol.

[8] Karsenty G, Ulmann A, Droz D, Carnot F, Grunfeld JP (1985). Clinical and histological resolution of systemic amyloidosis after renal cell carcinoma removal. Nephron.

[9] Vanatta PR, Silva FG, Taylor WE, Costa JC (1983). Renal cell carcinoma and systemic amyloidosis: demonstration of AA protein and review of the literature. Hum Pathol.

[10] Babu A, Lachmann H, Pickett T, Boddana P, Ludeman L (2014). Renal cell carcinoma presenting as AA amyloidosis: a case report and review of the literature. CEN Case Rep.

[11] Bazzi C, Petrini C, Rizza V, Arrigo G, D’Amico G (2000). A modern approach to selectivity of proteinuria and tubulointerstitial damage in nephrotic syndrome. Kidney Int.

[12] Gillmore JD, Hawkins PN, Pepys MB (1997). Amyloidosis: a review of recent diagnostic and therapeutic developments. Br J Haematol.

[13] Shimo T, Adachi Y, Yamanouchi S, Tsuji S, Kimata T, Umezawa K (2013). A novel nuclear factor κB inhibitor, dehydroxymethylepoxyquinomicin, ameliorates puromycin aminonucleoside-induced nephrosis in mice. Am J Nephrol.

[14] Lane T, Gillmore JD, Wechalekar AD, Hawkins PN, Lachmann HJ (2015). Therapeutic blockade of interleukin-6 by tocilizumab in the management of AA amyloidosis and chronic inflammatory disorders: a case series and review of the literature. Clin Exp Rheumatol.

[15] Okuda Y, Ohnishi M, Matoba K, Jouyama K, Yamada A, Sawada N (2014). Comparison of the clinical utility of tocilizumab and anti-TNF therapy in AA amyloidosis complicating rheumatic diseases. Mod Rheumatol.

[16] Okuda Y, Takasugi K (2006). Successful use of a humanized anti-interleukin-6 receptor antibody, Tocilizumab, to treat amyloid a amyloidosis complicating juvenile idiopathic arthritis. Arthritis Rheum.

